# Long-term Results of a Cardiology Postgraduate Program

**DOI:** 10.5935/abc.20170083

**Published:** 2017-06

**Authors:** Edimar Alcides Bocchi, Danielle Pazzotti Borges, Vagner Oliveira-Carvalho Rigaud

**Affiliations:** Instituto do Coração (InCor) - Faculdade de Medicina da Universidade de São Paulo, São Paulo, SP - Brazil

**Keywords:** Health Postgraduate Programs, Scientific and Technological Activities, Program Evaluation

## Introduction

Research and innovation help to drive economic growth and address socioeconomic challenges like poverty and health.^1^ Many developed and developing countries have introduced policies and systems to increase research and innovation.

In Brazil, a system was created on 1951 with the objectives of improving technology and innovation and strengthening scientific research.^2,3^ Similar to postgraduate doctorate programs in developed countries, postgraduate programs with broader and more in-depth scientific research objectives have been developed in Brazil.^4^ In fact, a linear relationship has been demonstrated between the number of students graduating from these programs and the number of scientific publications produced by them.^5^ Beyond scientific output, publishing a high-impact paper or in a journal with a high-impact factor seems to be an important requirement for innovation and technology growth. Considering that postgraduate students play an important role in scientific production in Brazil, a study including the characteristics of the scientific production of these students is justifiable.

We retrospectively investigated the scientific and academic production of students after their graduation from a cardiology postgraduate program. Because cardiovascular disease is the leading cause of death in developed countries and in Brazil, a postgraduate program focused on cardiology is a good target for innovation. Also, the knowledge of the characteristics, weaknesses, and strengths of a postgraduate program may help develop new strategies promoting innovation and publication in high-impact journals.

## Methods

The protocol of this study was submitted to our institution’s Ethics Committee on May 14, 2010, and received the number 3434/10/023. The Committee approved the study on December 15, 2010, with the number 385/10.

### Objectives

The primary objective of this study was to investigate the number of publications of each graduate of a cardiology postgraduate program in Brazil and the corresponding impact factor of the journals in which the graduates’ research was published.

The secondary objectives included the evaluation of the students’ characteristics, *h*-index, total citations, citations per article, and academic position.

### Study design

This was a retrospective study developed at *Instituto do Coração* (InCor), São Paulo. We defined as a graduate any postgraduate student obtaining a certificate at the end of the program between 1977 and 2010. The postgraduate program during the period of the study followed the rules set by the University of São Paulo for this type of program. The program was also evaluated from its beginning according to the criteria established by the *Coordenação de Aperfeiçoamento de Pessoal de Nível Superior* (CAPES, a Brazilian federal agency for the support and evaluation of postgraduate education). The students’ baseline characteristics used in this study were obtained at the time of the students’ registration in the program and included age, sex, and other data reported at baseline. These data were retrieved from the Cardiopulmonary Department program files in 2010.

A systematic review was carried out through a quantitative, retrospective, and documentary design for each student during the period that followed the completion of their postgraduate degree. The review included scientific papers published from 1977 to October 2015 and included in the Scopus and ISI Web of Science databases, as indicated by each postgraduate student in his or her Lattes curriculum. This curriculum is part of a Brazilian database created in 1999 and is supported by the *Conselho Nacional de Desenvolvimento Científico e Tecnológico* (CNPq) in which researchers may include information about their academic and scientific production (lattes.cnpq.br/). The name of each student was used for the review. The Lattes curriculum may also include data about the students’ affiliated institutions and research teams.

Scientific papers were excluded from the analysis if comprising abstracts, medical guides, technical and scientific reports, dissertations, ministerial and government information, or any other type of document not complying with the standard IMRDC structure (introduction, methods, results, discussion, and conclusion) applied to scientific papers, except for reviews, editorials, and comments addressing cardiovascular issues published in journals indexed in PubMed. Any article in which the student was the first author or a coauthor was included in the analysis.

### Statistical analysis

The data were statistically analyzed with GraphPad Prism 6 for Windows. The Shapiro-Wilk test was applied to verify the data’s Gaussian distribution. Descriptive statistical analysis included simple distribution of frequencies, calculation of proportions, and median and respective interquartile ranges (IQRs). Continuous variables are expressed as median and IQR, and categorical variables are expressed as percentage. For group comparisons, Mann-Whitney or Wilcoxon tests was used, when appropriated. All tests were performed 2-tailed, and a p level < 0.05 was considered indicative of statistical significance.

## Results

### Characteristics of the postgraduate students

**Figure 1:**
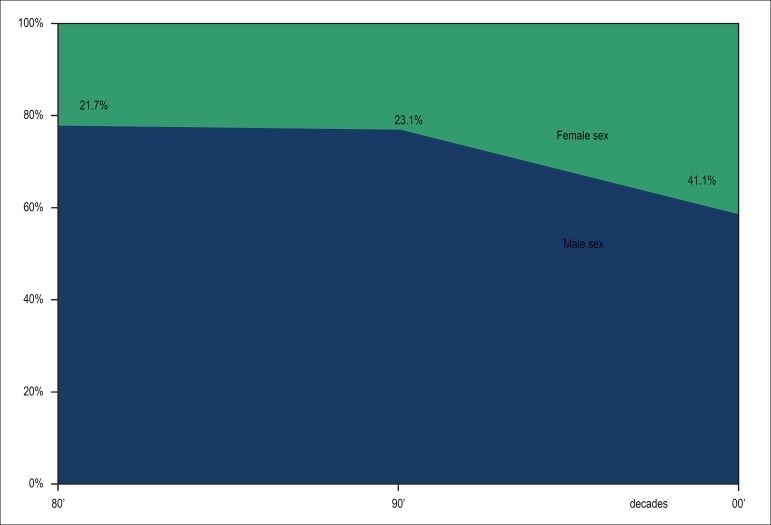
Gender distribution of the postgraduate students.

**Figure 2:**
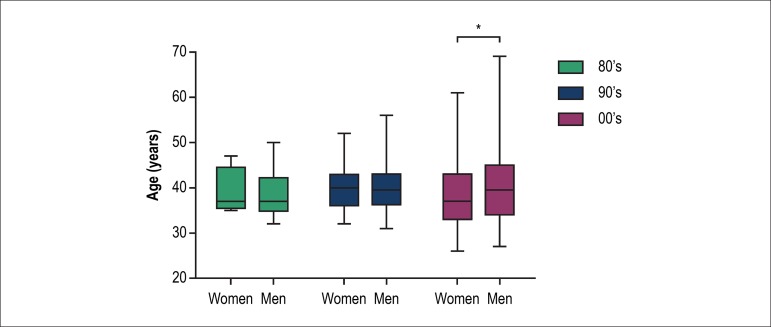
Mean age of the postgraduate students.

**Figure 3:**
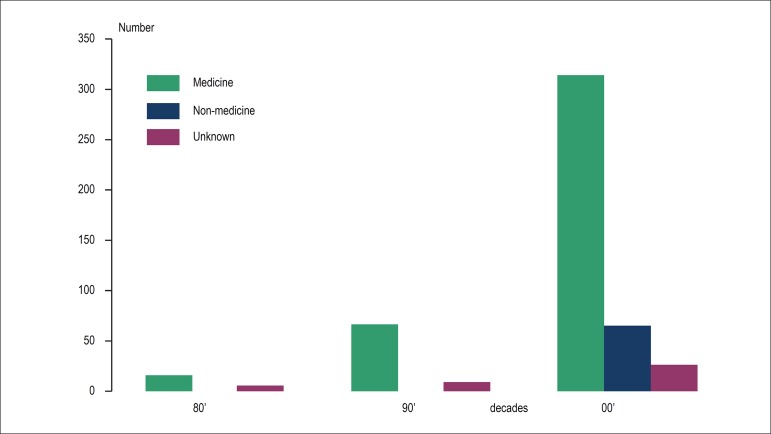
Postgraduate students with previous medical training versus no medical training.

The study included 505 students who had completed the postgraduate cardiology program. Most students were male, white, and had previously obtained a medical degree (Table 1). Figure 1 shows the recent incremental increase in women as postgraduate students in the cohort. The absence of the Afro-Brazilian ethnicity is remarkable in the student population, given the high numbers of Afro-Brazilians in the Brazilian population (Table 1). Most students had no prior master’s degree. Female students were younger than male ones, mainly in the last decade (Figure 2). The number of postgraduate students increased over the decades, and a recent increase in graduates without a medical degree was observed (Figure 3). We would also like to point out the low number of foreign students.

**Table 1:** Baseline characteristics of the postgraduate students

Variable	N (%) or median (IQR)
Total number	505 (100)
Male sex	316 (62.6)
Female sex	189 (37.4)
**Ethnicity**	
White	260 (51.5)
Afro-Brazilian	0 (0)
Mulatto	6 (1.2)
Yellow (Asian)	16 (3.1)
Ethnicity not provided	223 (44)
**Median age (all)**	
Female sex	37 (34-43)
Male sex	39 (35-44)
**Nationality**	
Brazilian	500 (99)
Non-Brazilian	5 (1)
**University graduation**	
Medicine	397 (78.6)
**Non-medicine**	
Biology	8 (1.6)
Biomedicine	8 (1.6)
Nursing	12 (2.4)
Electronic engineering	1 (0.2)
Pharmacy	5 (1)
Physiotherapy	5 (1)
History	1 (0.2)
Psychology	5 (1)
Nutrition	6 (1.2)
Chemistry	1 (0.2)
Veterinary	3 (0.6)
Physical education	6 (1.2)
Unknown	38 (7.5)
Previous master's degree	64 (12.7)
Ph.D. without previous master's degree	441 (87.3)

IQR: Interquartile range.

### Publications and corresponding impact of the publications’ journals

**Figure 4:**
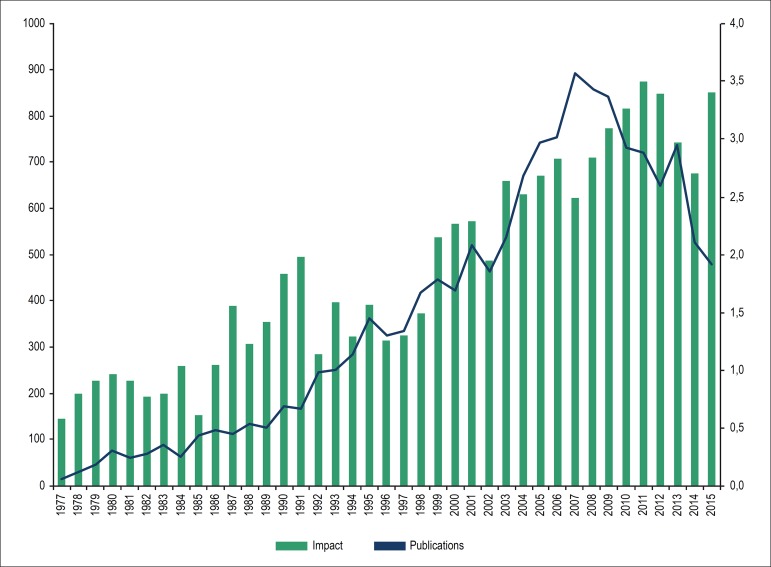
Number of publications per year by all postgraduate students and corresponding journal impact factors from 1977 to 2015.

**Figure 5:**
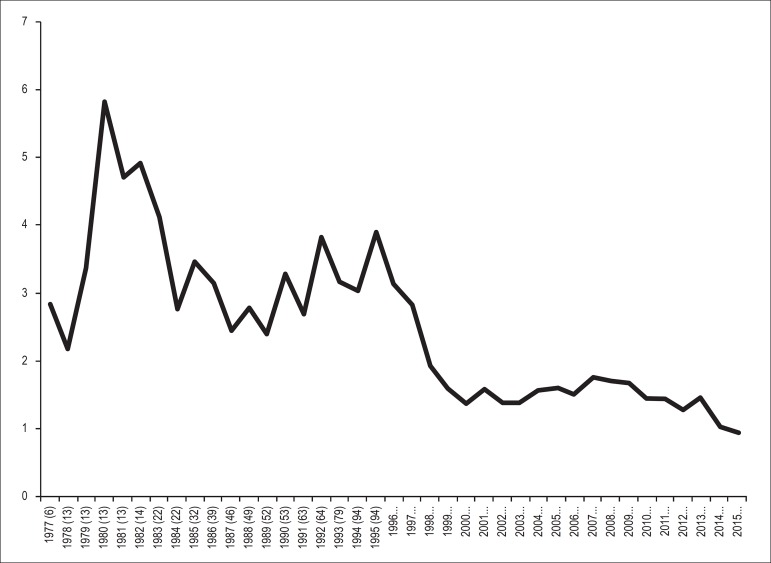
Number of publications from 1977 to 2015 adjusted for the number of postgraduate students with a theoretical capacity to publish.

From 1977 to October 2015, a total of 14,398 manuscripts were published in which the cardiology postgraduate students were first authors or coauthors. Figure 4 shows the number of publications per year by all postgraduate students and the impact factor of the journals in which the articles were published. A progressive increase in the number of publications may be observed until 2007, followed by a decrease from 2008 to 2015. The journals’ impact factors increased until 2011. Figure 5 shows the number of publications from 1977 to 2015 adjusted for the number of postgraduate students with a theoretical ability to publish. A decline in the number of publications may be observed from 1995 to 2000, after which it remained stable until 2013. A tendency towards a reduction in the number of publications may also be observed between 2014-2015.

**Figure 6:**
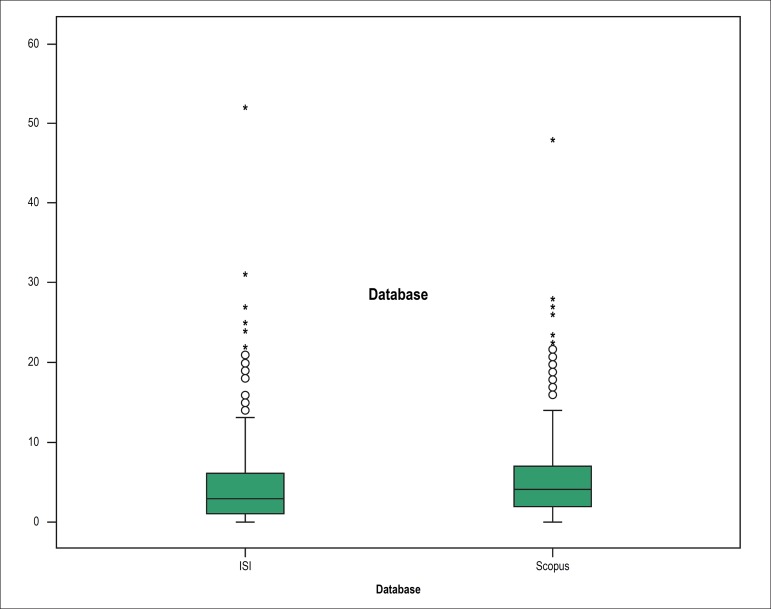
H-index values of the postgraduate students according to the ISI and Scopus databases.

Table 2 shows scientific indices and academic indicators related to the postgraduate students over the decades. The data show a small total number of articles published by year. The total number of citations was low, and the number of citations per article was not expressive. Likewise, the *h*-index was not high, according to the ISI and Scopus databases (Figure 6). Analysis of the *h*-index distribution revealed that 12.8%, 54.06%, 20.99%, 7.33%, 2.97%, and 2.57% of the students had *h*-index values of 0, 1-5, 6-10, 11-15, 16-20, and >20, respectively. University training in biology and biomedicine was associated with a lower *h*-index value and fewer published articles (Table 3). The median number of published articles and the *h*-index were higher among students with prior training in medicine (p < 0.0001 and p = 0.0042, respectively).

**Table 2:** Scientific indexes and academic indicators of the postgraduate students over decades after completion of the program

Scientific index	Scopus	ISI	Lattes
H-index	4 (2-7)	3 (1-6)	___
Published articles	10 (3-25)	7 (2-16.5)	13 (4-35)
Total number of citations	54 (11-244)	39 (5-167)	___
Citations per article	6 (2-12)	5.6 (2-12)	___
Published articles per year	1 (0.3-2.2)	0.6 (0.2-1.6)	1.5 (0.5-3.2)
Impact factor	___	___	1.5 (0.8-2.4)
**Academic indicators**			
Research			16%
University teaching			15.5%
Research and teaching			26.3%
Others			42.2%

**Table 3:** Scientific indexes and academic indicators of the postgraduate students according to the graduation program

Graduation	*H*-index		Impact factor	Articles
	**ISI**	**Scopus**		
Medicine	3 (1-6)	4 (2-6)	1.4 (0.8-2.5)	16.5 (5-40.3)
Non-medicine	2 (1-4.3)	2 (1-5)	1.8 (0.5-3)	4 (2-11.5)
Biology	1 (0-3)	3 (0-5)	2.9 (1-3.1)	3 (2-9)
Biomedicine	1.5 (0.3-2.8)	2.5 (0.5-3.8)	2.8 (1.2-3.3)	3.5 (2.3-9.3)
Physical education	4.5 (1-8.3)	4.5 (0-9)	1.8 (1.1-2.1)	15.5 (1.8-36.5)
Nursing	3 (2-5.8)	4 (1.3-5)	2.2 (0.7-3.7)	9.5 (5.8-14.5)
Pharmacy	4 (1-6)	4 (1-6.5)	3.5 (0.6-5)	8 (5-13.5)
Physiotherapy	1.5 (0.8-8.5)	2 (0.8-8.3)	1.2 (0.4-2.5)	5 (1.5-52)
Other	1.5 (0.8-3)	2 (0-3)	1.1 (0.2-3.1)	3 (1-5)

Following the end of the postgraduate cardiology program, only 42.3% of the students continued their research activities. Remarkably, 42.2% of the students did not follow research or teaching activities (Table 2).

## Discussion

To the best of our knowledge, this is the first study reporting the scientific output of graduates from a cardiology postgraduate program in Brazil. Our findings are relevant because cardiovascular disease is the most frequent cause of death in some developing and developed countries.^6^ The graduates of the largest cardiology program in Brazil had a progressive incremental in the total number of publications until 2007, mainly as a consequence of the expansion of the community of researchers. Also, the articles were published in journals with progressively higher impact factors until 2011, but these impact factors may be considered low. Moreover, the number of publications adjusted by the number of students reduced until 2000 and remained stable afterward. We observed that the scientific output per student was not homogeneous. The *h*-index, number of citations, and the number of publications of each graduate were poor. Only 42% of the graduates embraced research activities after the program, and the research they performed had a low impact. The population of postgraduate students also had special characteristics, including a low number of Afro-Brazilian students and foreigners, a progressive incremental rise in the number of students with a higher percentage of younger woman and students without prior medical training credentials.

Despite the success of the increase in the journals’ impact factors until 2011 and the total number of publications until 2007 (which declined as the number of graduate students increased), the scientific productivity by cardiology postgraduate students and its impact are concerning. The heterogeneity of the scientific production was also worrisome because it seems to have followed the Pareto principle, in which a minority is responsible for the greater part of the production. Brazilian scientific publications have increased significantly in number, but the citation indices have remained at approximately 60% of the world’s mean citations (Thomson Reuters). Despite this fact, the performance of Brazilian researchers is high among some developing and emerging countries.^7^ In a comparison with other countries, a recent bibliometric analysis demonstrated that the number of cardiovascular publications from Latin America increased from 1999 to 2008.^8^ Brazil was the country with the greatest increase in the number of publications. However, the citation index by year of publication in Brazil was 9 in 1999 and 9.1 in 2008, while in Argentina, this index increased from 9.2 to 25.6. The causes of poor scientific and academic output by cardiology postgraduate students are complex and largely unknown. Unfortunately, we lack published data from other postgraduate courses for the purpose of comparison. Many factors could be hypothesized to explain our findings. Although they might be interconnected, two periods can be considered to explain the causes of our findings: the training period for research during the postgraduate program, and the time after the program. During the training period in the postgraduate program, the initial module is provided to a potentially future researcher, whereas after the conclusion of the program, the student faces a real-world research scenario.

The cardiology postgraduate program was developed according to guidelines developed by CAPES, which may have influenced the training period of the program. CAPES has established criteria for the development of programs, measuring the scientific output of graduates from postgraduate programs and imposing goals for these individuals. The current CAPES criteria for evaluation of postgraduate programs in Brazil were initially established in 1998.^9^ The evaluation of each program is currently complex and includes an appraisal of the program’s proposal, faculty, students, intellectual output, and social inclusion. For the evaluation of the program, the impact of the scientific journals in which the articles are published is measured by a specific national index called *periódicos Qualis*. The Qualis system is an imperfect solution that considers the importance of the article according to the journal in which it is published, regardless of the number of citations.^7^ This evaluation criterion has never been validated prospectively and raises many concerns. Instead of focusing on strengthening scientific bases, technology, and innovation, CAPES has developed other objectives, such as the postgraduate training of teachers of all education levels and training of qualified human resources personnel for the non-academic market. Therefore, the rules established by CAPES may stimulate the training of more but low-impact cardiology researchers.

In addition to the rules established by CAPES, the postgraduate program is also influenced by the university’s environment. The university’s postgraduate board supports high-impact research, but this is actually not a top priority of the cardiology postgraduate program in the real world.^10^ One important factor seems to be the form of the final assessment of the scientific production of each postgraduate student. Rather than assessing the work done during the postgraduate program through the impact of its publications or the impact of the peer-reviewed journal in which the article was published, the evaluation is performed through a panel of local professors. As a confirmation of this fact, the rate of disapprobation of the theses presented as part of the program is almost nonexistent. In some situations, the publications are accepted for approval but are hindered by bureaucratic complexities. For example, the university’s postgraduate committee points out innovation as one of the objectives of the program but prioritizes other objectives instead, such as the teaching of training, leadership skills, and knowledge of the study field to postgraduate students. In addition, Brazilian universities have low classifications in international rankings, and this low ranking does not provide an enabling environment for high-impact research.^11^ Some other characteristics of the postgraduate program may contribute to that, such as a scenario of low-risk taking, lack of proper environment for boldly innovative ideas, no priority for innovation in the real world, submission of a research protocol before research training courses, attempt to prepare students for high-impact research using low-impact training, absence of environment or time for revolutionary or innovative ideas or high-impact research, lack of training by international researchers, replication of science rather than development of original science, and necessity of publication as early as possible regardless of the impact that such publication will obtain. In fact, after an analysis of the criteria and objectives established by CAPES and the universities, one might assume that high-impact publications and innovation are not the highest priorities of these institutions in the real world, and the methods used by them are not enough to secure publication in high-impact journals.^9,10^ Additional factors to explain the finding that high-impact research in the real world is not a priority for Brazilian universities are some lingering distortions from the French school model with its historical professional origin, institutions not integrating teaching and research, elitist attitude,^12^ and threat to creativity perceived by the privileged model because of the generation of new values as a consequence of innovations and technology. The persistence of remnants of the cathedral structure without consideration of merits for career growth also hinders high-impact scientific accomplishments.^13^

Regarding the time after the program completion, the national scenario of research institutions is not attractive for cardiology students in terms of the development of a research-oriented career and does not contribute to retaining research talent. Many factors may contribute to that, such as a historical culture lacking research encouragement, low income, accomplishments not properly recognized, the necessity of multiple jobs to obtain adequate income, and promotion of scientific and academic career and choice of leaders not based on merit.

The limited research resources offered by the government and private initiatives,^14^ the type of distribution of these resources, characteristics of the funding agencies, definitions of priority without enough social scientific transparency, and controversial criteria for the selection of the research to be supported may all influence cardiology graduates during the training period and after the completion of the postgraduate program. Unfortunately, high-impact research, with rare exceptions, is expensive. The popularity of providing research funds with low monetary value is contrary to high-impact research that results in innovation. Also, the low investment in research by private companies in Brazil is remarkable.

To worsen this scenario, foreign companies and institutions have developed in Brazil competitive and financially supported clinical research originating from other countries (without a “local technological value”) generating unfair competition with local, unfunded original research. Unfortunately, this type of research is generally designed in foreign countries without a true Brazilian authorship, and the Brazilian researchers participating are therefore subordinated. At the most, Brazilian researchers may secure the position of coauthors without becoming main authors. This may contribute to local laboratory discoveries remaining in what has been termed as the "valley of death” - a gap between bench research and clinical application.^15^ Additionally, there is not a critical mass of high-impact researchers acting in funding agencies as peer reviewers who can choose high-impact projects.

In general, the priorities and application of funds from funding agencies are not socially and scientific transparent. The lack of upgrading in funding agencies hinders them from rapidly adapting to new required strategies, considering that these agencies do not make bids for boldly innovative ideas. A cultural change is necessary for agencies considering innovation as a risky activity frequently not resulting in success. However, low investment in research and funding may not be enough to explain the low impact of the publications. In fact, the budget of the Brazilian Ministry of Science, Technology, and Innovation (MCTI) doubled from 2005 to 2010, but this fact was not associated with proportional relevant increments in publication impact.^16^ The current decrease in research investment following the 2014 economy stalling in Brazil is worrisome. One might suggest that Brazil is a “young” country with regards to research, which could explain the country’s limitations. However, other similarly young countries in terms of research, such as South Korea and China, have found success in innovation.^17^

The expectations of the cardiology postgraduate student also are important for low-impact publication, because the purpose of the program may sometimes be to complete and refine a previous learning deficiency mainly in research development and interpretation. Also, independently of a research career, graduates with a diploma from a postgraduate program will have better professional opportunities.

Finally, access to publishing in high-impact journals may have undisclosed obstacles, as such journals may prefer to publish manuscripts originating from developed countries. Research developed by Brazilian authors also has a low rate of true international collaboration. Some Brazilian researchers have attempted to overcome this limitation with the inclusion of foreign researchers without a well-defined international cooperation; fortunately, this is not a widespread procedure. Of note, articles with at least one foreign author may attract more citations.^7^ It has been recently reported that the country from where an article originates affects the perception of the article’s quality and relevance.^18^ Thus, Brazilian researchers may be compelled to publish in Brazilian journals without a high international prestige, therefore without attracting many citations.^7^ The median impact factor of most Brazilian journals is below those of thematic fields under international indexes.^7^ A vicious circle or Matthew effect could be influencing this scenario.

### Limitations

Since this retrospective study was conducted in the cardiology field, the internal validity of its results could be considered applicable only for a population of graduates of a cardiology postgraduate program. However, the finding that Brazilian publications have a low impact factor and the important role of the Brazilian postgraduate system in increasing the number of Brazilian publications are evidence of an external validity of our findings, at least in the medical area of cardiology. In other medical areas, the same low impact may be verified.^19^ On the other hand, it is possible that select postgraduate programs may have different characteristics and, consequently, diverse results.

Much of the Lattes curricula data were included by the graduates themselves; therefore, they could not be entirely verified. Excellent articles, mainly on the areas of Tropical Medicine and Public Health, are not accepted in foreign journals, especially articles considered of "regional interest." Then, extremely important information is oftentimes not properly propagated because the information is not considered as a "universal science."

In contrast, some researchers probably have their research impact increased by participating as coauthors in international trials without resulting in Brazilian innovation or contribution to national technological development (absence of creation of Brazilian value). In fact, an unacceptable disproportion between first authorship and coauthorship can be verified. Moreover, some researchers are not necessarily considered among those with ideas or innovative initiatives, and they often play a supporting role, albeit not a major one, in the research.^20^ Culturally, it may happen in Brazil, although uncommon, the inclusion of coauthors based on honor (in which the coauthors had no active participation in the research), either because of their hierarchical position at the institution where the research was performed, or for their referral of patients to the study, which is not compliant with the guidelines of the International Committee of Medical Journal Editors.^21^ The evaluation of the increased impact of the journals in which all scientific research was published may have limitations due to the historical increase in the number of journals in which cardiology articles are generally published.

We did not investigate the number of downloads of each article, which is being increasingly used to assess a publication’s impact. However, download statistics may have limitations. The number of downloads is not offered by most journals and may also include counts derived from search engine crawlers and downloads by non-scientific individuals. Therefore, the number of citations by other articles currently remains the gold standard for evaluation of the impact of an individual scientific article. Also, controversial results have been published concerning the correlation between the number of downloads and citations.^22,23^

Finally, we did not evaluate the publications’ economic output, including patents, device approvals, and value created. However, considering the low-impact of these publications, positive findings in this area are unlikely. Other variables, such as the *h*-index of the study advisor, appear to be also important predictors of publication success.^24^

### Implications

In addition to policies designed to increase scientific production, strategies to increase high-impact publications targeting innovation warrant changes to cardiology postgraduate programs and the period following completion of the program. Similar to the philosophical dilemma of the chicken or the egg coming first, the components are integrated and interdependent, but urgent modifications involving many factors should be planned, including related to CAPES, university rules, funding agencies, and the country’s scenario. In fact, the postgraduate system should be reconsidered. Also, a better balance between scientific output and high impact should be obtained.

Other important decisions depend on whether the current cardiology model is cost-effective to the country in training students in research with the knowledge that less than half of the graduates will actually pursue research careers, even low-impact ones. The development of separate programs for high-impact research and teaching should be tested as an alternative. Advanced Medical Education Research and Innovation (MERI) units are an example.^25^ At the postgraduate level, content should be more innovative, as in the UK.^26^

The assessment of academic and scientific output by graduates should be mandatory and extended to all postgraduate programs. In the evaluation criteria, scientific output by graduates should be required.

## Conclusion

The Scientific output of graduates should be considered in the evaluation criteria of postgraduate programs. Policies for access to socially vulnerable students and international students should be encouraged. Despite the success in increasing the total number of publications, the current proposed mechanisms to increasing publication in high-impact journal through this current postgraduate system seem to be ineffective. Our findings showing a low scientific output from graduates of a cardiology postgraduate program in regards to the low number of publications, impact factor, and *h*-index values warrant modifications in postgraduate programs’ plans, funding agencies, and the country’s scenario for research.
